# Active polypeptide MDANP protect against necrotizing enterocolitis (NEC) by regulating the PERK-eIF2ɑ-QRICH1 axis

**DOI:** 10.1038/s41598-023-44194-4

**Published:** 2023-12-21

**Authors:** Jie Huo, Rui Zhang, Xinping Wu, Changchang Fu, Jinhui Hu, Xiaohan Hu, Wenqiang Sun, Zhenjiang Chen, Xueping Zhu

**Affiliations:** 1 Department of Neonatology, Children’s Hospital of Soochow University, No. 92 Zhongnan Street, Industrial Park, Suzhou, Jiangsu 215025 People’s Republic of China; 2 Department of Neonatology, Yangzhou Maternity and Child Health Care Hospital, Yangzhou, People’s Republic of China; 3 Neonatal Medical Center, Huai’an Maternity and Child Health Care Hospital, Xuzhou Medical University, Huai’an, People’s Republic of China

**Keywords:** Gastrointestinal diseases, Nutrition

## Abstract

The effect of MDANP effects on ER stress signalling not well known or elucidated. Endoplasmic reticulum (ER) stress plays a critical role in necrotizing enterocolitis (NEC) pathogenesis through the PERK-eIF2ɑ-QRICH1 axis. The present study aimed to explore the protective effects of MDANP in NEC development. Firstly, a function screening was designed to identify the candidate peptides in human milk, and then the identified peptides were validated in NEC patients. In vivo, NEC was induced in mice pups and divided into four groups: (1) control group, (2) NEC group, (3) MDANP + NEC group, and (4) NS + NEC group. In vitro, lentivirus-mediated QRICH1 silencing, was used to transfect NCM460 cell lines, then stimulated with LPS. After LPS stimulation, cells were treated with chemically synthesized MDANP, and the essential proteins in the QRICH1 signalling pathway in cells were tested and compared. After the small-scale screening, a peptide (SKSKKFRRPDIQYPDATDED) named MDANP was determined as the principal peptide. Its protective effect against NEC through inhibiting the expression of ERS key proteins and impeding the intestinal cells’ apoptosis was observed in the animal models. Furthermore, the inhibitive effect of MDANP on apoptosis of intestinal epithelial cells through modulating the PERK-eIF2ɑ-QRICH1 ERS pathway was also confirmed in vitro. Taken together, our data suggest that MDANP effectively ameliorates apoptosis in NEC through attenuating PERK-eIF2ɑ-QRICH1.

## Introduction

Necrotizing enterocolitis (NEC) is a harmful gastrointestinal tract (GI) disease in preterm infants, particularly those with very low birth weights (VLBW; < 1500 g)^1,2^. NEC premature infant mortality is high, up to 50% of NEC premature infants undergoing surgical intervention^3^. Previous research focused on the role of endoplasmic reticulum stress (ERS) in NEC, which is a severe acute intestinal injury in preterm infants^4,5^. Some studies have confirmed that inhibition of continuous ER stress can prevent intestinal cell apoptosis, which reduces intestinal permeability and mitigates damage to intestinal barrier function^6–8^.

There is evidence supporting the relationship between ERS and acute intestinal injury, thereby underscoring the importance of preventing ERS-induced apoptosis to inhibit the development of certain diseases, such as NEC^9,10^. Unfolded protein response (UPR) is a stress response that that allows cells to respond to endoplasmic reticulum stress. However, Paneth cells may cause dysfunction or apoptosis^11^. Steady state activation of UPR regulates and enhances the critical aspects of the entire secretory pathway. UPR signaling survives and eventually an alternative signaling program called "terminal UPR" has been developed. Finally, apoptosis is promoted^12,13^. In some cases, tissue inflammation can alter the steady state of cellular proteins accumulation of UPR in the ER. During endoplasmic reticulum stress (ERS) conditions, PKR-like endoplasmic reticulum kinase (PERK) gets activated and undergoes autophosphorylation, leading to its oligomerization. This process involves the phosphorylation of eukaryotic translation initiation factor-2 (eIF2α) and results in the inhibition of general protein translation^14^. The transcriptional regulator QRICH1 is a distinct arm of the PERK-eIF2a axis. Cells respond dynamically to ER stress by upregulation of QRICH1. The QRICH1 transcriptional procedure plays an important role in the management of cell stress response and the adaptive adaptation of cells to the access to UPR and terminal UPR^15^.

Human milk contains a series of microorganisms and bioactive ingredients that guide development of the infant mucosal immune system^16,17^. Bioactive peptides, an important component of human milk, exhibit a variety of functions such as anti-infection, immune regulation, and growth promotion^18,19^. Peptidomic analysis has revealed that many polypeptides are present in human milk^20^. Polypeptides with low molecular weight and acid and alkali resistance are not easily degraded in the intestine; are easy to synthesize, modify, and optimize; and generally have no or minimal side effects, making them of tremendous pharmaceutical value^21^.

Milk-derived anti-NEC peptide (MDANP) in human milk may protect against NEC by regulating this axis. The purpose of this study is to explore the protective effects of MDANP against NEC development.

## Materials and methods

### Establishment of neonatal mouse model of NEC

C57BL/6 mouse pups (age: five-day-old) were purchased from JOINN Laboratories Co., Ltd. (Suzhou, China). The animal experiment was approved by the Ethics Committee of the Soochow University (Approval No. 3348). The pups were separated from their mothers after birth and given lipopolysaccharide (LPS) (Sigma, US) (4 μg/g body weight, 3 times a day) by gavage. Allocation the formula of model of NEC:15 g of Similac Advance infant formula (Abbott Laboratories, Chicago, USA) and 75 mL of dog milk substitute (PetAg, Illinois, USA). The mice were fed five times per day through self-made gavage device feeding. Put the mouse pups into a nitrogen box (concentration of 5% O2, lasting for 10 min each time) to simulate a hypoxic environment, lasting for 10 min each time, and repeated four times a day. The modeling method was based on study by our research team^22^. In this study, all methods were carried out in accordance with relevant guidelines and regulations and followed the recommendations in the ARRIVE guidelines.

### Animal groups and treatments

All animals were randomly divided into four groups: (1) control, (2) NEC, (3) NEC + NS, and (4) NEC + MDANP groups, which received intraperitoneal injections of MDANP or NS.

### Peptide synthesis and treatment

During the early stage, we identified 85 differentially active polypeptides in the human milk of premature and healthy term infants with multiple NEC, and screened the polypeptides displaying strong mass spectrum signals, large differential multiples, and stable structures (named milk-derived anti-NEC peptides [MDANP]) to explore their biological functions. The carrier peptide of HIV1-TAT sequence (YGRKKRRQRRR) is covalently linked to the N-terminal of MDANP (SKSKKFRRPDIQYPDATDED), which is synthesized by Shanghai Science Peptide Biotechnology Co., Ltd. First, the peptide were dissolved in sterilized water with appropriate concentration and conjugated with fluorescein 4-isothiocyanate (FITC), 50 μM was added to the cell culture. The cell fluorescence was measured after 3 h to verify the ability of MDANP to cross the membrane. For in vivo animal studies, normal saline (NS) was used as the control.

### Cell culture and silencing QRICH1 in NCM460 cells

The NCM460 cell line, which originated from the human intestine (Procell Life Science and Technology Co., Ltd. ). NCM460 cells were cultured in Roswell Park Memorial Institute (RPMI) medium (10% fetal bovine serum (FBS), penicillin and streptomycin). The cells were stored in a incubator (37° C ,5% CO2). QRICH1 silencing in NCM460 cells was performed according to the manufacturer's instructions (GeneChem, Shanghai, China). Dividing cells into four groups: (1) control group without drugs; (2) Lipopolysaccharide (LPS) stimulated cells (Invitrogen, CA, USA); (4) cells pretreated with MDANP for 3 h and stimulated with LPS for 12 h; And (4) NEC + MDANP + QRICH1 shRNA cells transfected with QRICH1 shRNA were pretreated with MDANP for 3 h and stimulated with LPS for 12 h. LPS stimulated cell concentration in this experiment was 10 μg/mL. The infection magnification (MOI) is 10:1. Then the cells were infected with shRNA lentivirus particles (GeneChem, Shanghai, China) and mixed gently and incubated for 6 h, the cells were incubated with RPMI for 48 h. Subsequently, infected cells were cultured in RPMI medium containing 0.5 µg/mL purinomycin to select stable infected cells expressing QRICH1 shRNA.

### Reverse transcription quantitative polymerase chain reaction (RT-qPCR)

Complementary DNA (cDNA) was prepared using qScript cDNA SuperMix (Qiagen, Maryland, USA) and S1000 Thermal Cycles (Bio-Rad Laboratories, California, USA). Total RNA was extracted using TRIzol reagent (Thermo Fisher Scientific, Inc., CA, USA). RT-qPCR is performed using the advanced qPCR master mixing and CFX384 real-time system (Bio-Read Laboratories, Inc.). The cDNA and primers were used for quantitative PCR (qPCR) using SYBR Green Master Mix (Roche, Switzerland) and LightCycle 480 II equipment. The housekeeping gene GAPDH (glyceraldehyde-3-phosphate dehydrogenase ) is used. Normalize the data to GAPDH and use 2^−ΔΔCt^ method calculation^23^. The primers used in this study are shown in Table 1.

**Table 1:** The primers used in this study.

Gene	Species	Forward sequence (5′ to 3′ )	Reverse sequence (5′ to 3′)
Cleaved caspase-3	Human	GTGATAAAAGTAGAAGT	TGGAGAATAAAACCT
GAPDH	Human	GTCTCCTCTGACTTCAACAGCG	ACCACCCTGTTGCTGTAGCCAA
PERK	Mouse	GGGTGGAAACAAAGAAGAC	CAATCAGCAACGGAAACT
eIF2ɑ	Mouse	GATCTGGCCAGACGCCTTGA	CCTCCTCATCCTCCTCGTTCGAT
QRICH1	Mouse	GGCCAGGCACCACAGTCAGGCAGG	CTTTCCCCACCACCATCCCA
Cleaved caspase-3	Mouse	ATGGAGAATAAAACCT	CTAGTGATAAAAGTAGAAGTTC
GAPDH	Mouse	TGACCTCAACTACATGGTCTACA	CTTCCCATTCTCGGCCTTG

### Western blot analysis

First, intestinal tissues were homogenized using RIPA lysis buffer and proteins were extracted (intestinal tissues grinder model: KZ-III, Wuhan Servicebio Technology Co., Ltd.) add it into RIPA lysis buffer containing 1% protease inhibitor (Beyotime Institute of Biotechnology, China) for lysis, and then add the loading buffer (5 ×), boil for 10 min at 100 °C. Transfer the protein from the gel with polyvinylidene fluoride membrane (Millipore, Billerica, MA, USA), and membrane it with5% non-fat milk in Tris-buffered saline (TBS) containing 0.05% TBS-T buffer buffer for 1 h (at room temperature). After three times of membrane washing, the membrane was membraned with 5% bovine serum albumin (BSA), the membrane was cut before incubated with primary antibody at 4 °C on a shaking table overnight. Primary antibodies: anti-phosphorylated QRICH1 (1:1000; Sigma Aldrich), anti-PERK (1:2000; Abbkine), anti-phosphorylated eIF2 (1:1000, American cell signal), anti-cleaved caspase-3 (1:1000) and anti-GAPDH (1:5000; Abcam). Then the secondary antibody (1:5000; Abcam) conjugated with HRP was incubated at room temperature for 1 h. Use the biological image analysis system (Syngne, MD, USA) to use the ECL reagent (Beyotime Institute of Biotechnology) for immune detection and visualization. The band density was compared with that of endogenous reference protein GAPDH.

### Immunofluorescence assay

The blood on the surface of the intestinal tissue of mouse pups was washed with normal saline, and then the intestinal tissue was fixed with 4% paraformaldehyde, and 0.1% Triton x-100 was added into PBS for 10 min. After the blocking step, the tissue and primary antibody were incubated overnight at 4° C, and then incubated at room temperature with Alexa Fluor-coupled secondary antibody (Invitrogen) 1 h and then add DAPI (Vector Laboratories) incubate for 10 min. The staining results were observed using a fluorescence microscope (model:TZL-500, Suzhou Percival Experimental Equipment Co., Ltd).

### Statistical analysis

The experimental data are expressed as mean ± SD. The level of apoptosis and ERS marker mRNA and western blotting results were correlated with the average level of the control group. Student’s *t*-test was used to compare the differences between the two groups. It is believed that *p* < 0.05 is statistically significant. All data measurement and analysis were carried out by persons who were not informed of the experimental group. All experiments were repeated three times.

## Results

### MDANP decreases the severity of NEC in neonatal mouse

**Figure 1:**
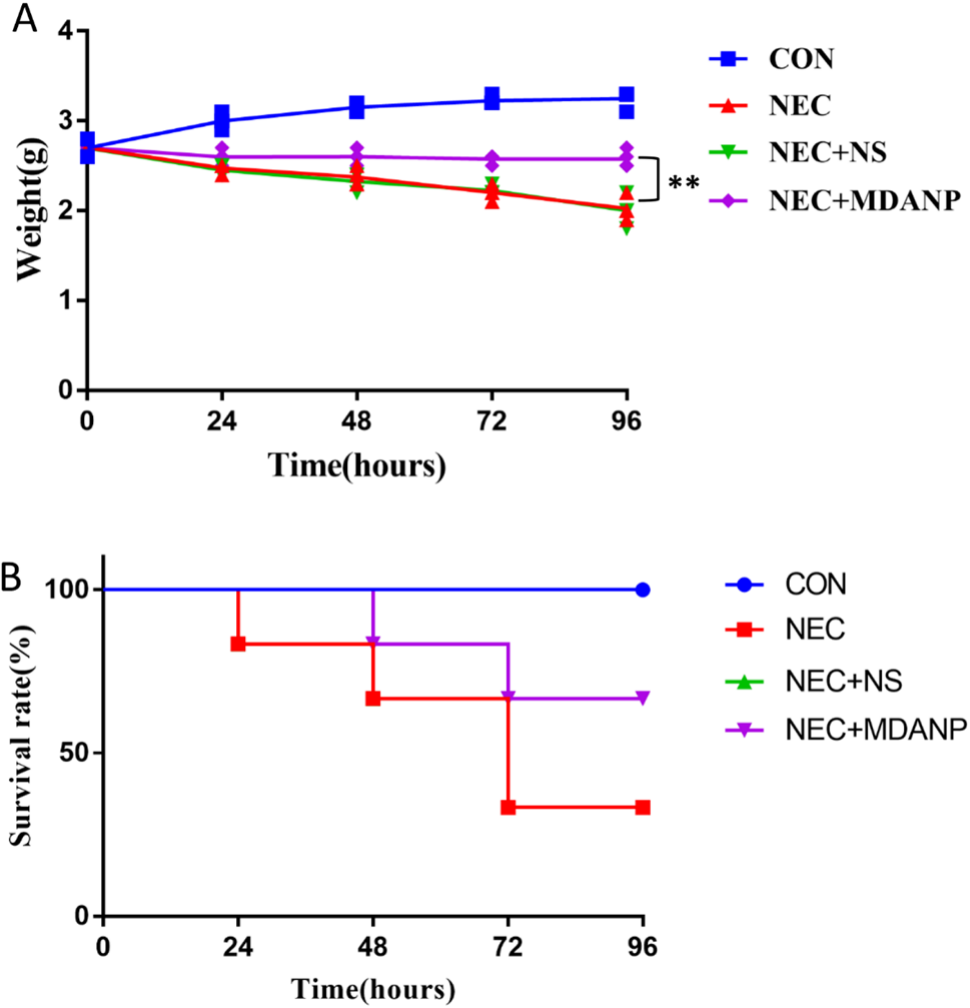
The effect of MDANPon body weight, survival. (**A**) Changes in the body weight of the surviving mice in each experimental group. (**B**) Kaplan–Meier survival analysis of the survival time, data are expressed as mean ± SD. NEC group. vs. NEC + MDANP group. **p < 0.01.

To verify the protective effect of MDANP intervention in neonatal mice. The body weight and survival rate of neonatal mice in each group were monitored. During the establishment of the model, the NEC induction group found that the weight of mice gradually decreased. Although MDANP treatment significantly reduced the weight loss induced by NEC, the body weight of NEC + MDANP group did not return to the weight level of CON group on the 3rd and 4th day (*p* = 0.002). There was no significant difference in body weight between NEC group and NEC + NS group (Fig. 1A). This study excluded technical death (not caused by the disease itself) and calculated the survival rate. It is worth noting that NEC group showed a lower survival rate (33.33%) 96 h after the establishment of NEC model. In contrast, MDANP treatment protected mice from NEC-related mortality and increased the survival rate of NEC + MDANP group to 66.67%; The survival rate of NEC and NEC + NS groups was 33.33% (Fig. 1B).

### MDANP ameliorates intestinal injury and protection in mouse NEC model

**Figure 2:**
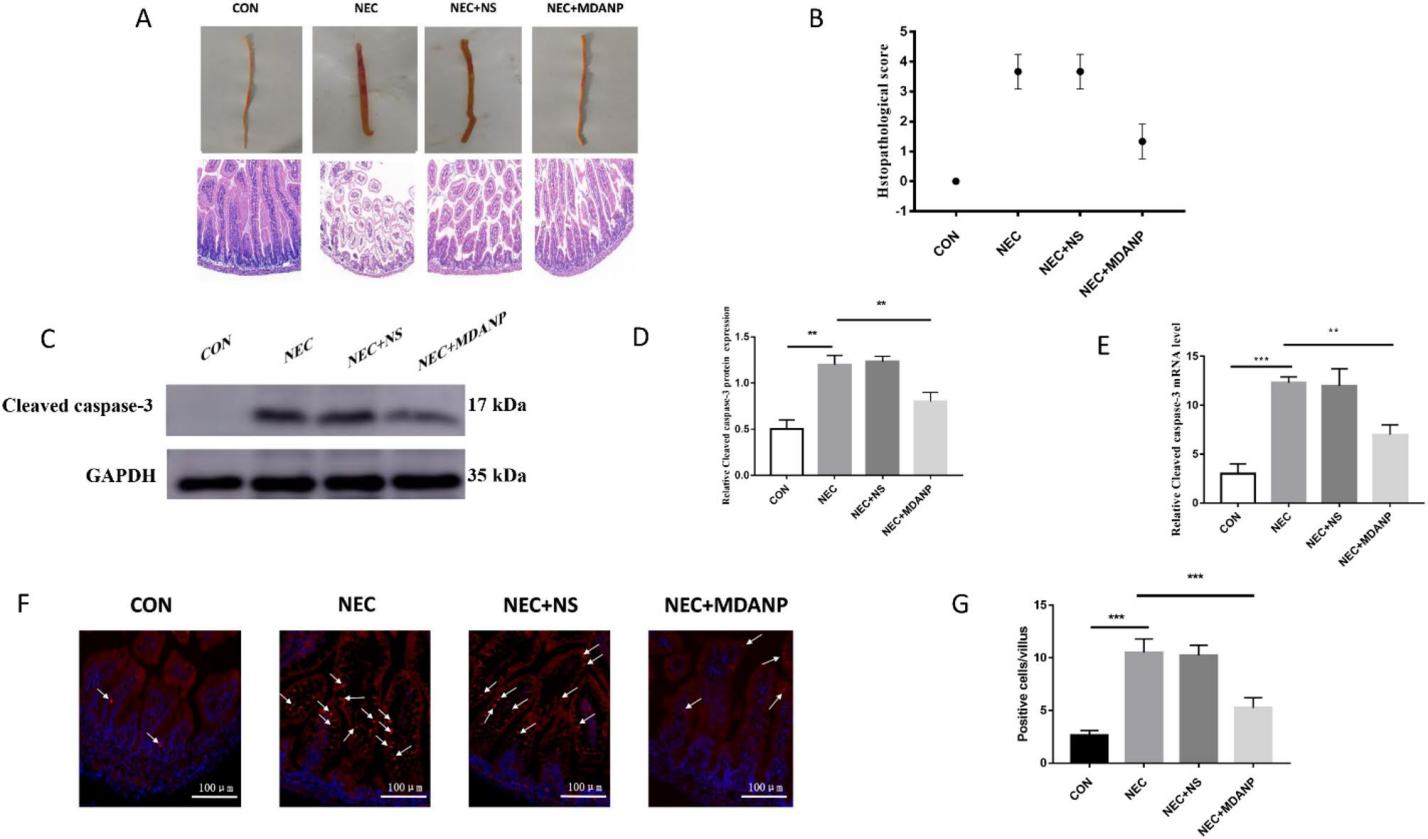
MDANP ameliorates intestinal injury and protection in mouse NEC model. (**A**), Representative histological images of the ileum of CON, NEC, NEC + NS, and NEC + MDANP mouse pups obtained by hematoxylin and eosin staining. Scale bars are equivalent to 100 µm in the images shown. (**B**), Histological slides were graded by two blinded investigators based on the NEC histopathological scoring system (*p* < 0.05). (**C**–**E**), Western blot and q-PCR analysis of the protein and mRNA expression levels of Cleaved caspase-3. (**F**), Representative images of immunofluorescence staining of apoptotic marker cleaved caspase 3 (CC3) and (**G**) quantification of immunofluorescence of apoptotic marker cleaved caspase 3 (CC3) for the listed groups. Scale bars are equivalent to 100 µm in the images shown. White arrow indicates the positive staining.

In vivo, we established an NEC animal model to test the effects of MDANP. Five-day-old mice were selected for gavage of formula milk, and hypoxia was simultaneously induced for 4 days. These behaviors lead to inflammatory cell infiltration, loss of surface epithelium, and epithelial defects beyond the mucosal muscle layer. Interestingly, mice fed with MDANP only showed NEC stress due to mild intestinal mucosal injury (Fig. 2A). The histopathological score^24^ of the NEC + MDANP group was higher than that of the control group, but lower than those of the NEC and NEC + NS groups (Fig. 2B). Simultaneously, we measured the proapoptotic protein CC3 levels in the intestine. The relative mRNA level in the NEC + MDANP group was lower than that in the NEC group, indicating that apoptosis was significantly reduced (Fig. 2C). Here, we measured the expression level of the CC3 protein in the mouse colon. The level of CC3 protein in NEC mice increased significantly and decreased slightly after treatment with MDANP, indicating that intestinal cell apoptosis improved (Fig. 2D). MDANP treatment also decreased NEC-induced apoptosis, as shown by the downregulation of CC3 (Fig. 2E,F,G). These observations indicated that MDANP improved NEC intestinal injury and reduced apoptosis.

### MDANP effectively penetrates cells

**Figure 3:**
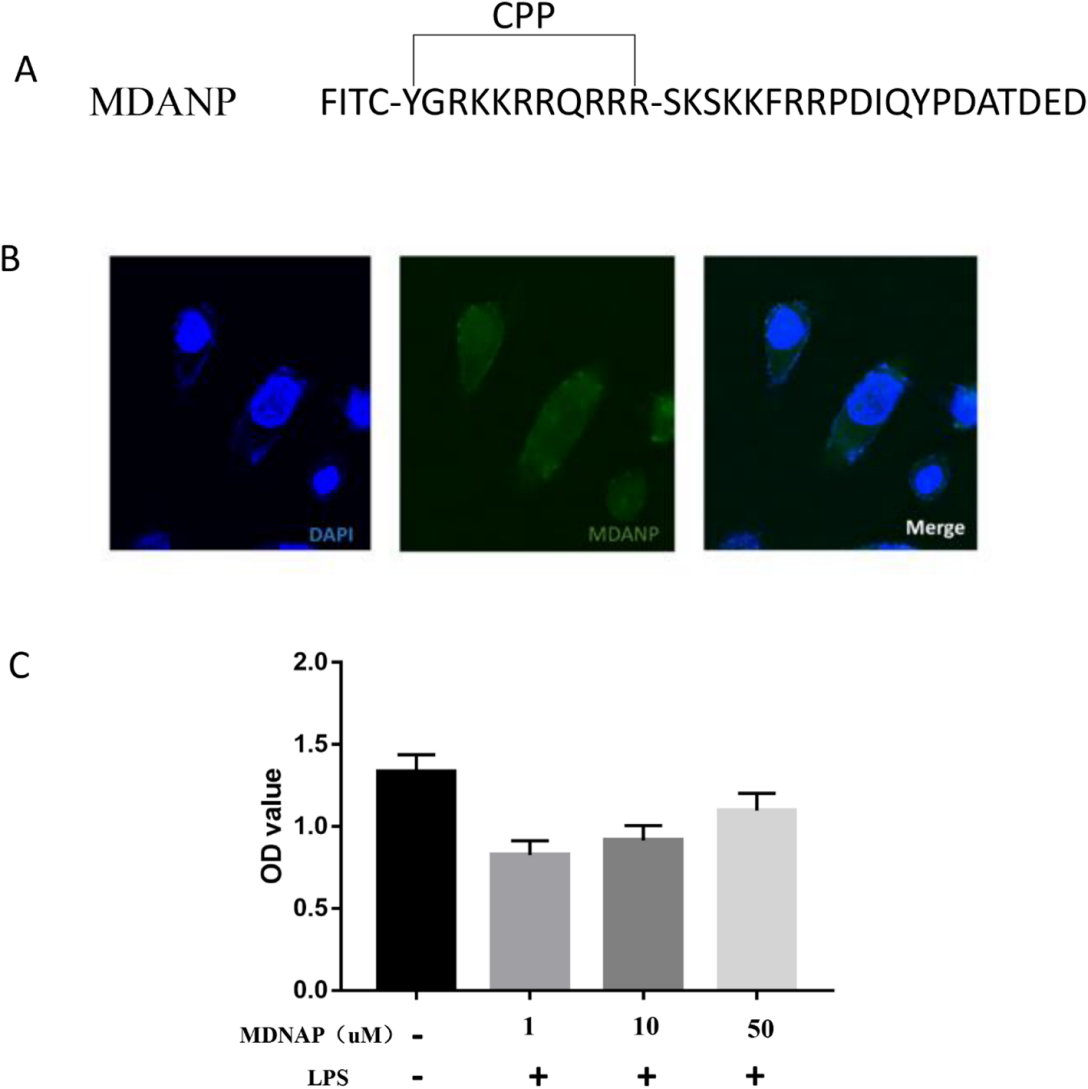
MDANPcould penetrate into cells effectively. (**A**) The sequence of synthesized MDANP. (**B**) FITC‐labeled APTBP (50 μM) were adding to the culture medium of FHC cells for 3 h and the cells were stained with DAPI and then photographed through phase‐contrast microscopy and fluorescence microscope. (**C**) The ODD of addition MDANP. Magnification: × 20. MDANP: Milk-Derived Anti-NEC Peptides; DAPI: 4′,6‐diamidino‐2‐phenylindole; FITC: fluorescein isothiocyanate.

The MDANP were obtained or synthesized by Shanghai Science Peptide Biological Technology Co., Ltd. To promote its entry into cells, MDANP was synthesized with an N-terminus attached to a cell-penetrating peptide (CPP, HIV-1 peptide (47–57), YGRKKRRQRRR; Fig. 3A). We also synthesized fluorescein isothiocyanate (FITC)-labeled MDANP to dissolve the peptide in sterile water and added it to NCM460 cell culture medium. We observed an distinct distribution of fluorescence in the cytoplasm of FITC-MDANP 3 h after administration (Fig. 3B). As shown in the CCK-8 experiment, the viability of MDANP-stimulated cells was significantly reduced; however, the viability of LPS-stimulated cells improved after the addition of MDANP (50 μM) (Fig. 3C). All the original protein imprinting images are included in the Supplementary Information file.

### MDANP attenuates ER stress through PERK-eIF2 α- QRICH1 pathway in NEC mice

**Figure 4:**
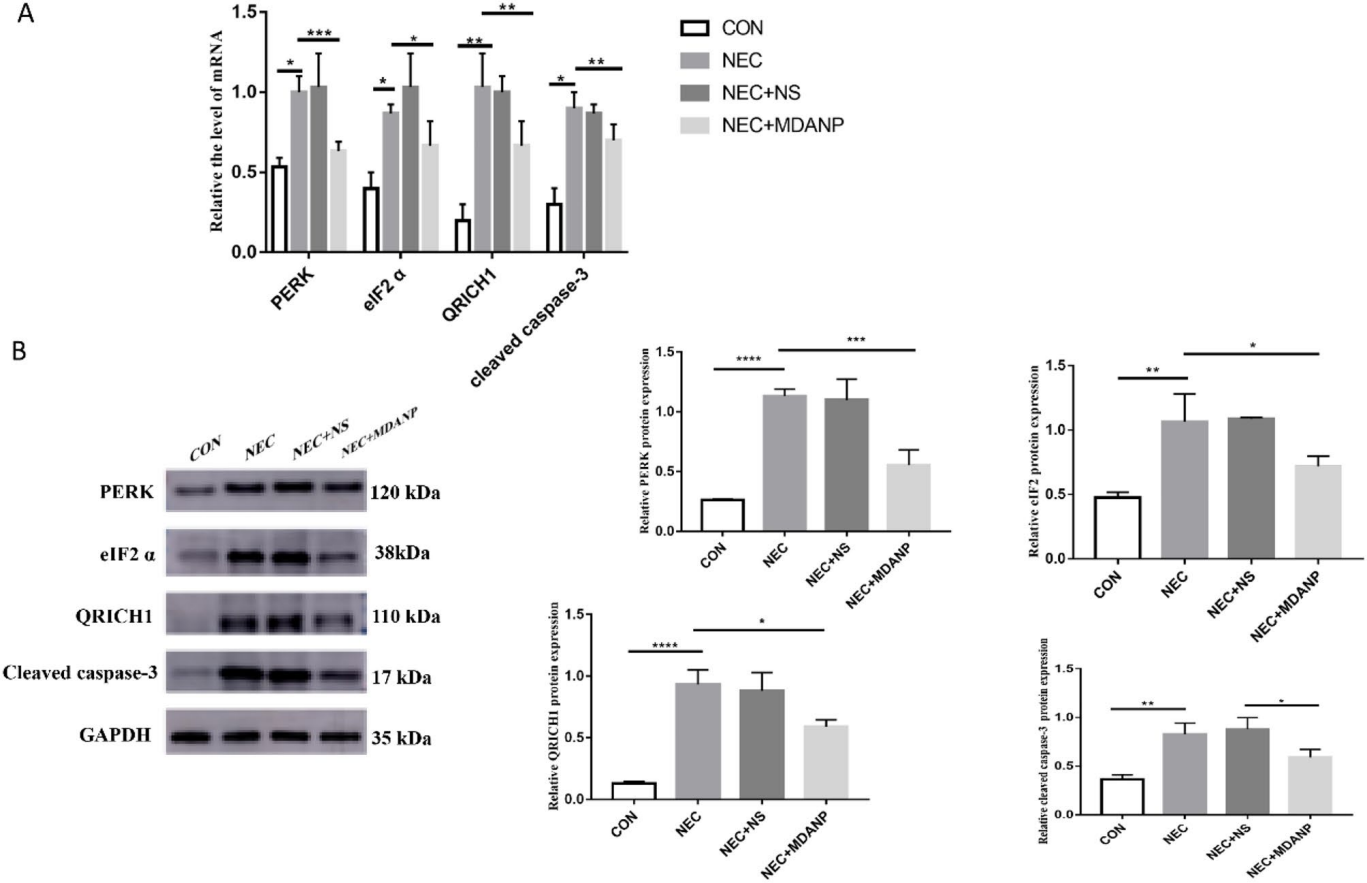
The effect of MDANP on ERS-related proteins in a mouse model of NEC. (**A**) NEC + MDANP group the mRNA levels of cleaved caspase 3 decreased. (**B**) Western blot analysis of the expression levels of PERK, eIF2α, QRICH1, cleaved caspase 3 and GAPDH proteins in the ileocecal part of intestinal tissue of mice among four groups. Data are expressed as mean ± SD for three independent experiments.**p < 0.01 and ***p < 0.001.

To investigate the protective effect of MDANP against ERS-induced apoptosis, we measured the protein and mRNA expression of CC3 in animal tissues. qRT-PCR and western blotting results revealed that after treatment of NEC mice with MDANP, the mRNA levels of CC3 decreased (Fig. 4A). To further investigate how MDANP improves ERS, the protein levels of PERK, p-eIF2, and QRICH1 were measured in vitro. Western blot results indicated that treatment with MDANP (1 mg/kg) reduced the levels of PERK, p-eIF2, and QRICH1 proteins (Fig. 4B).

### Silencing QRICH1 shows that MDANP attenuates apoptosis via the PERK-eIF2 α- QRICH1 pathway in NCM460 cells

**Figure 5:**
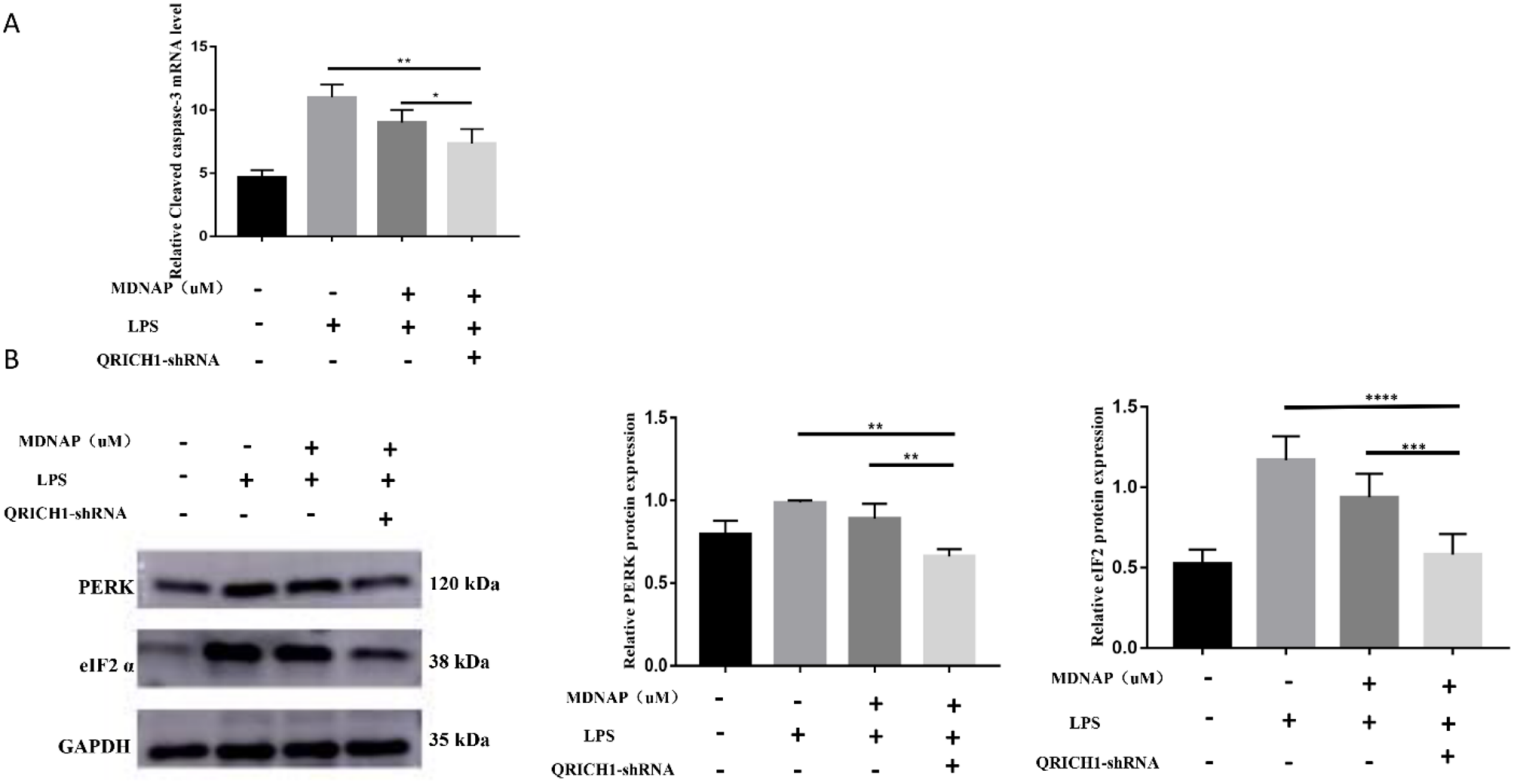
LPS stimulated NCM460 cells, and QRICH1 silencing mediated apoptosis of NCM460 cells through PERK-eIF2 α- QRICH1 pathwa**y.** (**A**) The mRNA expression of cleaved caspase 3 decreased in QRICH1-shRNA after MDANP treatment. (**B**) Compared with NEC + MDANP + QRICH1 shRNA group to NEC + MDANP group, the expression of ER stress related proteins such as PERK and eIF2a decreased. The results shown were observed in at least three independent experiments*p < 0.05, ** p < 0.01, ***p < 0.001.

In order to continue to study the potential role of MDANP in apoptosis, QRICH1 shRNA was used to silence NCM460 cells exposed to LPS stimulation. We found that the mRNA expression of cleaved caspase 3 decreased in QRICH1-shRNA after MDANP treatment compared to that CON group and LPS stimulated group (Fig. 5A). Silencing QRICH1 gene can weaken ERS, as shown by Western blot of NCM460 cells stimulated by LPS. We found that the NEC + MDANP + QRICH1 shRNA group had low protein expression levels of PERK and eIF2a (Fig. 5B). These results indicate that MDANP plays a protective role and may reduce apoptosis through the PERK-eIF2 α- QRICH1 pathway.

## Discussion

Although the diagnosis and treatment of premature infants have improved with the improvement of medical standards, the incidence rate and mortality of NEC have not significantly decreased^19^. NEC is the most common life-threatening gastrointestinal disease encountered in infants with VLBW(very low birth weight)^25^. However, its pathogenesis is not completely clear. Our present study revealed that MDANP can improve intestinal injury and reduce intestinal epithelial cell apoptosis in neonatal mice with NEC. The mechanism underlying MDANP-mediated inhibition of intestinal cell apoptosis may involve regulation of the PERK-eIF2ɑ-QRICH1 ERS pathway. Our previous study explored whether the occurrence of NEC is related to different polypeptides in human milk. Our current results reveal that MDANP can improve intestinal damage in NEC.

Polypeptides often combine with proteins to regulate the activity of target proteins to perform biological functions, Our previous research using PredictProtein predicted that MDANP has three potential protein binding sites. We explored whether MDANP has beneficial effects in preventing NEC both in vivo and in vitro. Studies have demonstrated that breastfeeding is the most effective way to protect newborns from NEC, although the precise underlying mechanism remains unclear. Increasing research into polypeptides may shed light on this issue. Previous studies reported that the pathology leading to NEC can occur in intestinal epithelial damage and necrosis induced by endoplasmic reticulum stress^26^. In our study, MDANP decreased the mRNA expression and protein content of apoptotic proteins, thereby protecting the integrity of the intestinal epithelium, which may be due to a reduction in the protein load^27^.

Proteomic analysis has confirmed that there are many types of polypeptides in human milk^20^. It has been reported that even peptides consisting of only 3 amino acids in breast milk can play an important active role^28^. The polypeptide itself has the advantages of low molecular weight, tolerance to acid and alkali resistance, difficult degradation in the intestinal tract, easy synthesis, modification, and optimization, and generally has no or minimal side effects; it thus has tremendous medicinal value^29^. Our study also observed that MDANP (50 µ M) can quickly enter the cell, and fluorescence distribution was detected in the cytoplasm and nucleus after 3 h of administration.

The maladaptive ER stress response may be related to several pathological conditions. The up-regulation of QRICH1 promoted UPR-mediated proteotoxicity. In addition, it was found that QRICH1 was significantly up-regulated in inflammatory intestinal epithelial cells of UC patients^15^. QRICH1 can maintain the stability of protein, and the imbalance of QRICH1 transcriptional activity may increase the sensitivity of intestinal epithelial cells to endoplasmic reticulum stress in the process of inflammation. The study also confirmed that knocking down QRICH1 can inhibit ERS, thereby reducing intestinal injury. The increase of ER stress can be used as an early marker of NEC intestinal injury. Our research also confirmed that ER stress has an important relationship with the occurrence of NEC.

Although the ability of MDANP to regulate endoplasmic reticulum stress has been confirmed, we want to determine whether MDANP also plays a role by interacting with some target proteins. Future studies, such as co-immunoprecipitation, are required to explore candidate targets and clarify detailed mechanisms (Supplementary Figure 1).

Taken together, our study revealed that the human milk constituent MDANP can protect from NEC by regulating endoplasmic reticulum stress in intestinal cells and weakening the apoptosis of intestinal cells. Our study provides new clues for the discovery and development of more effective therapeutic drugs for NEC. Exploring and optimizing its stability, targeting, and drug formation, facilitating the conversion research of MDANP for clinical treatment of NEC in the later stage.

## Conclusion

MDANP is a novel active peptide that we have screened and identified from human milk, which has the function of improving intestinal cell apoptosis and thus has a protective effect on NEC. Our research shows that MDANP can improve intestinal injury in NEC, which may ameliorate apoptosis in NEC through attenuating PERK-eIF2ɑ-QRICH1 axis. When human milk is unavailable, the use of synthetic peptides may become an alternative treatment measure to prevent NEC in newborns.

### Supplementary Information

Supplementary Figures.

## Data Availability

The data for this study can be obtained by contacting the corresponding author.
