# Supplementary material for: Active polypeptide MDANP protect against necrotizing enterocolitis (NEC) by regulating the PERK-eIF2ɑ-QRICH1 axis
Source: Sci Rep. 2023 Dec 21;13:22912. doi: 10.1038/s41598-023-44194-4 (PMC10739873; doi:10.1038/s41598-023-44194-4)
Supplement: Supplementary file 1 — Supplementary Figures. [file 41598_2023_44194_MOESM1_ESM.pdf]

cleaved caspase 3 (CC3)

GAPDH

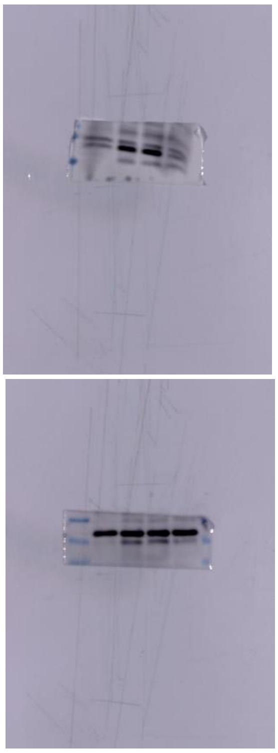

Fig 2C

PERK

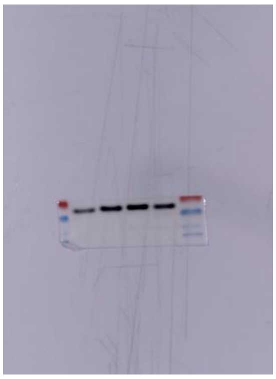

eIF2 α

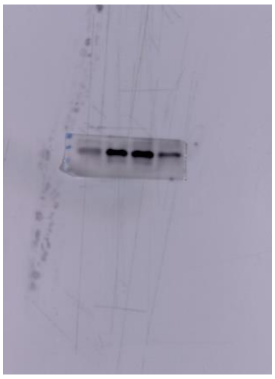

QRICH1

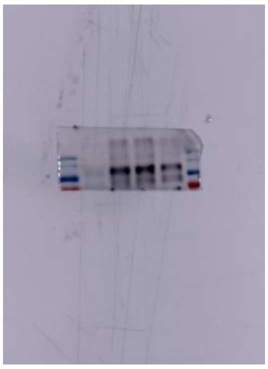

Cleaved caspase 3 (CC3)

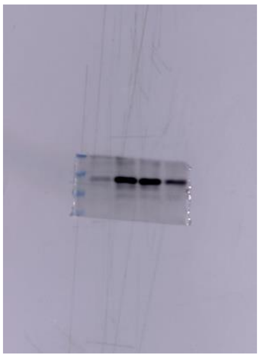

GAPDH

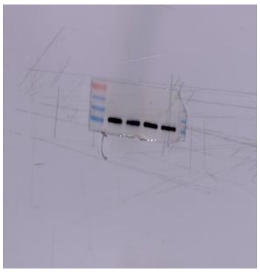

Fig4B

PERK

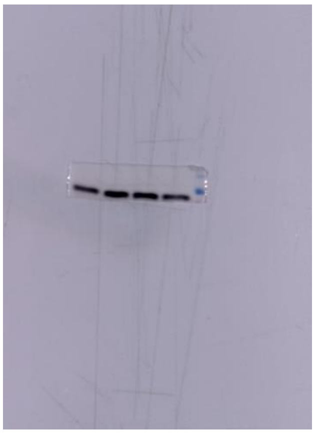

eIF2 α

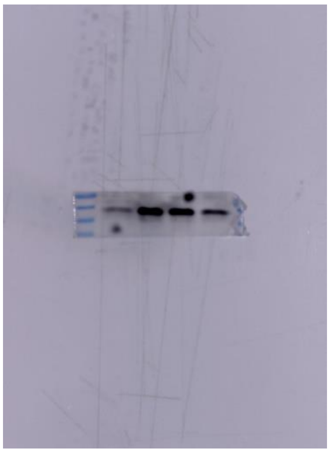

GAPDH

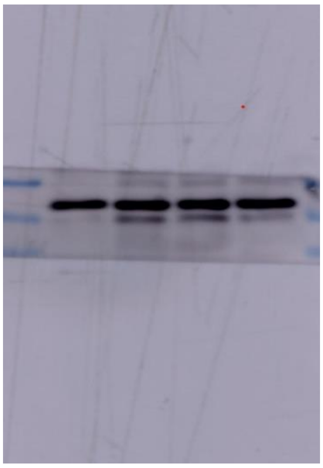

Fig 5B
